# A case series of profilometric changes in two implant placement protocols at periodontally compromised non-molar sites

**DOI:** 10.1038/s41598-021-81402-5

**Published:** 2021-01-18

**Authors:** Kwantae Noh, Daniel S. Thoma, Jung-Chul Park, Dong-Woon Lee, Seung-Yun Shin, Hyun-Chang Lim

**Affiliations:** 1 Department of Prosthodontics, School of Dentistry, Kyung Hee University, Seoul, Republic of Korea; 2 Clinic of Reconstructive Dentistry, University of Zurich, Zurich, Switzerland; 3 Department of Periodontology, College of Dentistry, Dankook University, Cheonan-si, Republic of Korea; 4 Department of Periodontology, Veterans Health Service Medical Center, Seoul, Republic of Korea; 5 Department of Periodontology, Periodontal-Implant Clinical Research Institute, School of Dentistry, Kyung Hee University, 26 Kyungheedae-ro, Dongdaemun-gu, Seoul, 02447 Republic of Korea

**Keywords:** Dental diseases, Dentistry, Therapeutics

## Abstract

Information regarding profilometric changes at a soft tissue level following implant placement with different protocols is insufficient. Therefore, this study aimed to comparatively investigate the profilometric tissue changes with respect to late implant placement following alveolar ridge preservation (LP/ARP) and early implantation (EP) in periodontally compromised non-molar extraction sites. Sixteen patients were randomly assigned to the following groups: implant placement 4 months post-ARP (group LP/ARP) and tooth extraction and implant placement 4–8 weeks post-extraction (group EP). Dental impressions were obtained immediately after final prosthesis insertion and at 3, 6, and 12 months. At the time of implant placement, bone augmentation was performed in the majority of the patients. Profilometric changes of the tissue contour were minimal between the final prosthesis insertion and 12 months in the mid-facial area (0.04–0.35 mm in group LP/ARP, 0.04–0.19 mm in group EP). The overall tissue volume increased in both groups (1.70 mm^3^ in group LP/ARP, 0.96 mm^3^ in group EP). In conclusion, LP/ARP and EP led to similar stability of the peri-implant tissue contour between the final prosthesis insertion and at 12 months. Moreover, the change of peri-implant tissue on the soft tissue level was minimal in both modalities.

## Introduction

Despite a vast amount of research regarding dental implants, there appears to be a gap between clinical research and practice. The main concern is the inclusion criteria in clinical trials. Due to standardization and consistency, healthy patients and healthy sites have been predominantly recruited for investigating the various implant timing protocols. However, clinicians are confronted with a relatively high number of compromised sites in daily practice^1^.

Implant placement protocols are defined based on the time point of implant installation after tooth extraction. A recent European Workshop in Periodontology defined the timing of implant placement with respect to tooth extraction^2^: (1) immediate placement (immediately after tooth extraction), (2) early placement (at 4–8 weeks), (3) delayed placement (at 12–16 weeks), (4) conventional (late) placement (> 16 weeks), and (5) delayed or late placement modified with alveolar ridge preservation (ARP). One can reasonably assume that each protocol has a different baseline due to the healing dynamic of the extraction socket. Moreover, the degree of the bone wall destruction in the extraction socket may influence the healing, potentially affecting the implant timing of choice.

Considering the above, the extent of further hard and/or soft tissue augmentation procedures and changes of the ridge profile (contour) can significantly depend on the implant placement protocols. However, limited comparative data exist regarding different implant placement protocols^2,3^. The following comparisons were mainly made: immediate vs. late placement^4,5^ and late placement with ARP versus without ARP^6,7^. Depending on the comparisons, different parameters were designed in each study, leading to insufficient information in some parameters. Specifically, information regarding the changes of the ridge profile and comparison of this parameter between different implant placement protocols is insufficient.

In a previous study, we compared soft tissue levels, periodontal parameters, and patient-reported outcome measures between late implant placement following ARP (group LP/ARP) and early implant placement (group EP) for periodontally compromised non-molar extraction sites^8^. In that study, no significant differences were noted in any of the parameters. However, three-dimensional profilometric changes on the soft tissue level over time have not been analyzed. To confirm the stability of the implant protocols, the profilometric changes should be further scrutinized.

The null hypothesis of the present study was as follows: one of the implant placement modalities (group LP/ARP or group EP) would not differ from the other one in terms of the linear (primary outcome) and volumetric changes on the soft tissue level between the prosthesis insertion and 12-month follow-up for periodontally compromised non-molar extraction sites. For this purpose, the tissue surface geometry was comparatively evaluated using the superimposition of Standard Tessellation Language (STL) files from dental impressions between implant prosthesis insertion and 1 year thereafter.

## Materials and methods

### Study design

In the present study, the data additionally obtained from the previous randomized clinical trial^8^ were analyzed. The original study protocol was approved by the Institutional Review Board (IRB) of Kyung Hee University Dental Hospital, Seoul, South Korea (KHD IRB 1511-2, approval date: November 20, 2015). A brief information about such study is presented in Supplement 1. In order to use the data for additional three-dimensional analyses using STL files from dental casts, the revised protocol was reapproved by the IRB (KHD IRB 1511-2, approval date: August 29, 2019). The experimental methods in the present study fully followed the reapproved study protocol.

Due to a limited research body about the comparison between implant placement protocols, the present study is characterized as an explorative study.

### Study population

Patients were recruited between March 2016 and January 2017 in the Department of Periodontology, Kyung Hee University Dental Hospital, Seoul, South Korea. Informed consents were obtained from all patients by designated investigators. The following inclusion and exclusion criteria were applied:

#### Inclusion criteria

Patients (1) aged ≥ 20 years, (2) with appropriate oral hygiene for dental surgery, and (3) with non-molar tooth requiring extraction and replacement with a dental implant were eligible for the present study. Only extraction sockets with ≥ 3 mm of hard and/or soft tissue loss in one or more socket walls^8^ and not more than 75% loss of the buccal bone plate were included for selecting periodontally compromised extraction sites.

#### Exclusion criteria

Patients with (1) heavy smoking habit (≥ 10 cigarettes per day), (2) uncontrolled systemic diseases, (3) untreated periodontal disease, (4) pregnant patients, (5) patients undergoing head and neck radiation, (6) patients with systemic conditions and receiving medications affecting soft and hard tissue healing, and (7) patients with alcoholism and drug addiction were excluded.

### Study groups

- Group LP/ARP: late implant placement following 4 months of healing post-ARP
- Group EP: early implant placement (4–8 weeks following tooth extraction)

### Surgical procedures

#### Tooth extraction/alveolar ridge preservation

After injecting local anesthesia (lidocaine containing 1:100,000 epinephrine), the buccolingual flaps were reflected. Gentle tooth extraction with degranulation was performed. Immediately after the tooth extraction, the envelope containing random group assignment was opened by an assistant to identify the assigned groups.

In group EP, a cross suture was used for flap approximation. In group LP/ARP, a deproteinized bovine bone mineral containing 10% collagen (DBBM-C; Bio-Oss Collagen; Geistlich, Wolhusen, Switzerland) was gently filled in the extraction socket. For deficient socket walls, DBBM-C was placed to create a homogenous contour with the neighboring ridge. Subsequently, a native bilayer collagen membrane (NBCM; Bio-Gide; Geistlich) was placed to extend at least 1–2 mm over the defect margin and socket entrance. An additional layer of NBCM was placed around the socket entrance^9^. The flaps were closed using crisscross and/or interrupted sutures. Primary flap closure was not attempted. After 7–10 days, the sutures were removed.

#### Implant placement

After 4–8 weeks, implant placement and guided bone regeneration (GBR) using DBBM (Bio-Oss; Geistlich) and NBCM (Bio-Gide; Geistlich) were performed in group EP. Adequate primary stability was obtained in all implants (Dentium, Seoul, Korea). A slight over-contour was made using DBBM particles compared to the adjoining ridge. Subsequently, NBCM (Bio-Gide; Geistlich) was placed to completely cover the grafted area. Primary flap closure was achieved using mattress and interrupted sutures. After 10–14 days, the sutures were removed.

At 4 months post-ARP, implant placement was performed in group LP/ARP. Additional bone augmentation with DBBM (Bio-Oss; Geistlich) and NBCM (Bio-Gide; Geistlich) was planned for the thin buccal bone left after the implant placement (< 1 mm) or in case of buccal dehiscence/fenestration defects. When bone augmentation was performed, the primary flap closure was made. Otherwise, a healing cap was connected to the implant for non-submerged healing. After 10–14 days, the sutures were removed.

In the case of bone augmentation, healing caps were connected after 3–5 months of healing. Soft tissue augmentation was not performed in any case.

After the surgeries, antibiotics and analgesics were prescribed to patients for 7 days. The patients were instructed to rinse with a 0.12% chlorhexidine solution twice a day until their sutures were removed.

### Follow-up

**Figure 1:**
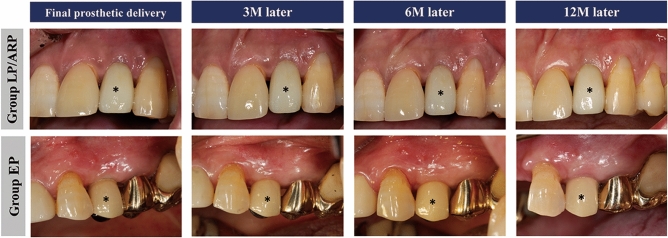
Clinical photographs over time. Asterisk indicates the implant crown included in the present study.

After 4–6 weeks following abutment connection, the patients were referred to the Department of Prosthodontics, Kyung Hee University Dental Hospital, Seoul, South Korea, for prosthetic treatment. Cemented- or screw-retained fixed prosthesis was inserted according to prosthodontists’ preference. The patients were recalled on the day of the final prosthesis insertion (T0) and after 3 (T3), 6 (T6), and 12 months (T12) for maintenance and data acquisition (Fig. 1).

### Outcome measures

Alginate impressions were obtained at T0, T3, T6, and T12. The impressions were completely covered with wet gauze. After waiting the elastic recovery of alginate impression (approximately 10 min), dental stone casts were made within 1 h. The stone casts were optically scanned with a three-dimensional model scanner (Rainbow Scanner Prime; Dentium, Seoul, Korea). The scanned data were saved as STL files. The digital analysis was performed by an experienced prosthodontist (K.N.). For each patient, the STL files obtained at T0, T3, T6, and T12 were imported into a computer software (Geomagic Control X; 3D Systems; Rock Hill, USA). To superimpose the surface models, a best-fit algorithm was utilized. The implant crown surface of each case was used as a reference. Two types of analyses were performed as follows:

#### Linear changes

**Figure 2:**
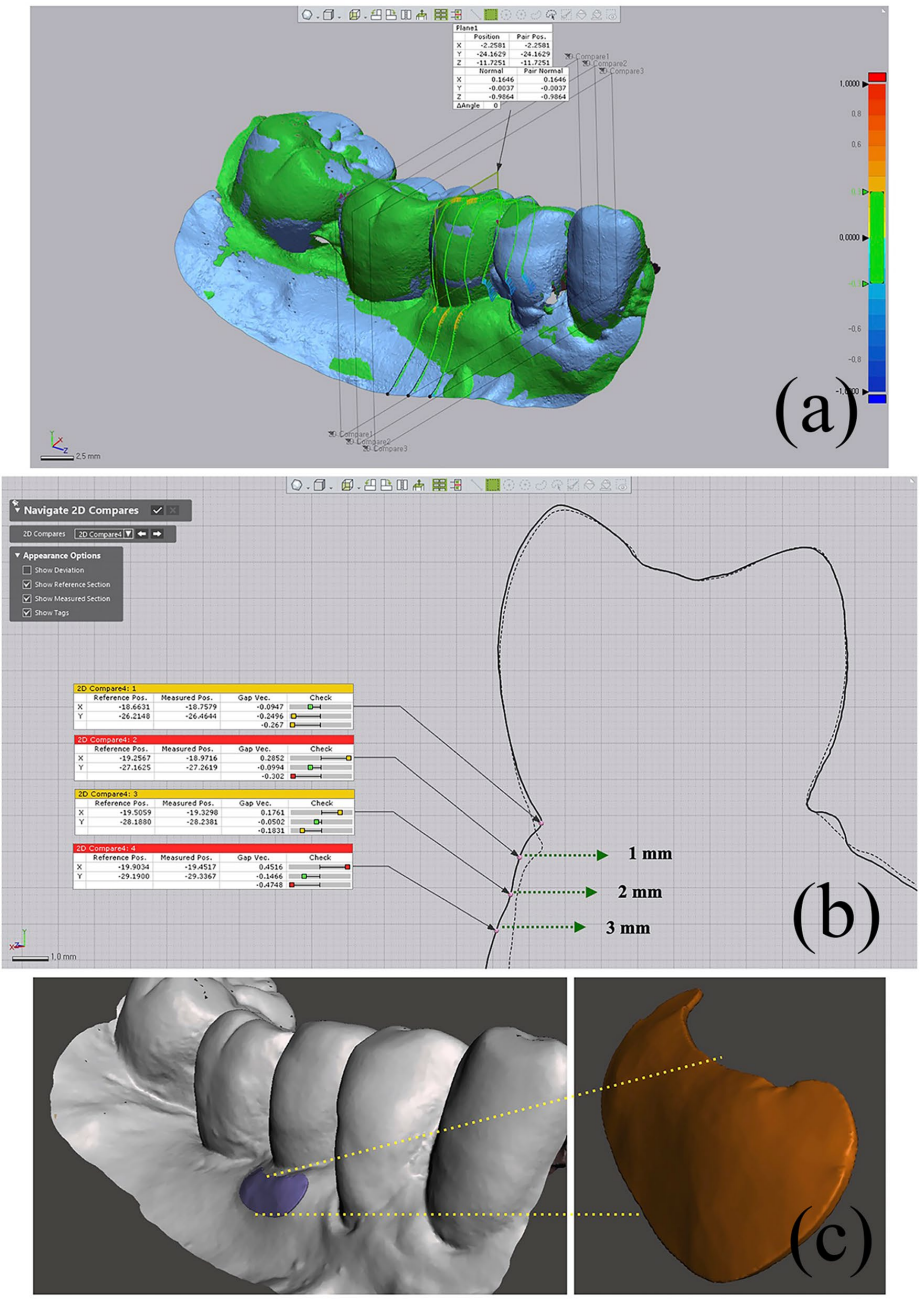
Linear and volumetric measurements. (**a**) The linear difference between the two time points was calculated in the mid-facial lines and mesial/distal line angles. (**b**) The difference was calculated at 1-mm, 2-mm, and 3-mm levels below the mucosal margin. (**c**) The volume difference was calculated in the set area of interest. The following are the reference points: 1 mm and 3 mm below the mucosal margin of the implant crown, extension lines of mesial and distal line angle lines.

A reference line along the long axis of the implant crown was made mid-facially. Subsequently, three points were marked at the 1-mm, 2-mm, and 3-mm levels below the mid-facial and mesial/distal line angle of the mucosal margin of the implant crown (Fig. 2a, b).

#### Profilometric changes

The area of interest (AOI) was defined using the following references: 1 mm and 3 mm below the mucosal margin of the implant crown, extension lines of the mesial/distal angle lines. Due to individual differences in the implant crowns, the size of the AOIs varied (Fig. 2c).

Subsequently, linear and volume differences between T0 and T3, T0 and T6, and T0 and T12 were calculated.

### Statistical analyses

Data are expressed as a mean ± standard deviation, median, and interquartile range. Due to the small sample size, nonparametric Mann–Whitney U tests were performed to examine the statistical difference between the two groups (Statistical Package for the Social Sciences statistics version 20.0; IBM, Armonk, N.Y., USA). A *p* value of < 0.05 was considered statistically significant.

## Results

The demographic data are presented in Table 1. Twenty-three patients were enrolled, but two in group LP/ARP and five in group EP were dropped out (due to refusal, change of treatment plan, and protocol violation). Finally, nine and seven patients in groups LP/ARP and EP were included in the analysis, respectively. Further bone augmentation procedures were performed in 7 of the 9 patients in group LP/ARP and in all 7 patients in group EP. No implant failure and no biologic complications were observed during the 12 months following final prosthesis insertion.

**Table 1:** Demographic information.

	Group LP/ARP (*n* = 9)	Group EP (*n* = 7)
Age (years)	58.11 ± 7.77	58.57 ± 15.57
Male/female	8/1	3/4
Mandible/maxilla	2/7	3/4

Group LP/ARP: late implant placement following 4 months of alveolar ridge preservation, Group EP: early implant placement.

### Linear changes

#### Changes from immediately after final prosthesis insertion (T0) and after 3 months (T3)

At the mid-facial area, the median contour increased at all levels in group LP/ARP (ranging between 0.12 mm [Q1: − 0.03, Q3: 0.26] and 0.28 mm [0.08, 0.45]), whereas in group EP, the median contour decreased (ranging between 0.02 mm [− 0.05, 0.27] and − 0.17 mm [− 0.22, 0.09]) (*p* = 0.681, *p* = 0.174 and *p* = 0.210 at 1 mm, 2 mm and 3 mm level, respectively). Positive and negative numbers indicate gain and loss, respectively.

#### Changes from immediately after the final prosthesis insertion and after 6 months (T6)

At the 1-mm level on the mid-facial area, a loss in contour was observed in both groups (− 0.08 mm [− 0.12, 0.28] in group LP/ARP and − 0.14 mm [− 0.22, 0.04] in group EP). In all comparisons between the groups, there was no statistically significant difference observed (*p* = 0.408, *p* = 0.470 and *p* = 0.091 at 1 mm, 2 mm and 3 mm level, respectively) (Table 2).

**Table 2:** Linear tissue changes at the mid-facial area on the soft tissue level.

Time point	T3–T0		T6–T0		T12–T0	
Group	LP/ARP	EP	LP/ARP	EP	LP/ARP	EP
1 mm	0.06 ± 0.21	− 0.05 ± 0.26	0.04 ± 0.26	− 0.1 ± 0.25	0.06 ± 0.26	− 0.01 ± 0.21
	0.13 (− 0.17, 0.18)	− 0.17 (− 0.22, 0.09)	− 0.08 (− 0.12, 0.28)	− 0.14 (− 0.22, 0.04)	0.04 (− 0.05, 0.26)	0.04 (− 0.18, 0.15)
*p* value	0.681		0.408		0.408	
2 mm	0.13 ± 0.16	0.05 ± 0.23	0.12 ± 0.18	0.04 ± 0.23	0.14 ± 0.23	0.1 ± 0.19
	0.12 (− 0.03, 0.26)	− 0.07 (− 0.09, 0.16)	0.05 (0, 0.22)	− 0.02 (− 0.09, 0.16)	0.16 (− 0.01, 0.21)	0.07 (0.06, 0.13)
*p* value	0.174		0.470		0.758	
3 mm	0.25 ± 0.21	0.15 ± 0.31	0.32 ± 0.19	0.1 ± 0.23	0.33 ± 0.25	0.24 ± 0.19
	0.28 (0.08, 0.45)	− 0.02 (− 0.05, 0.27)	0.26 (0.19, 0.41)	0.07 (− 0.02, 0.22)	0.35 (0.12, 0.52)	0.19 (0.11, 0.36)
*p* value	0.210		0.091		0.408	

Data are presented as a mean ± standard deviation, median (1st quartile, 3rd quartile). Positive and negative numbers indicate gain and loss, respectively. LP/ARP: late implant placement following 4 months of alveolar ridge preservation, EP: early implant placement. There were no statistically significant differences between the two groups at all levels.

#### Changes from immediately after the final prosthesis insertion and after 12 months (T12)

**Figure 3:**
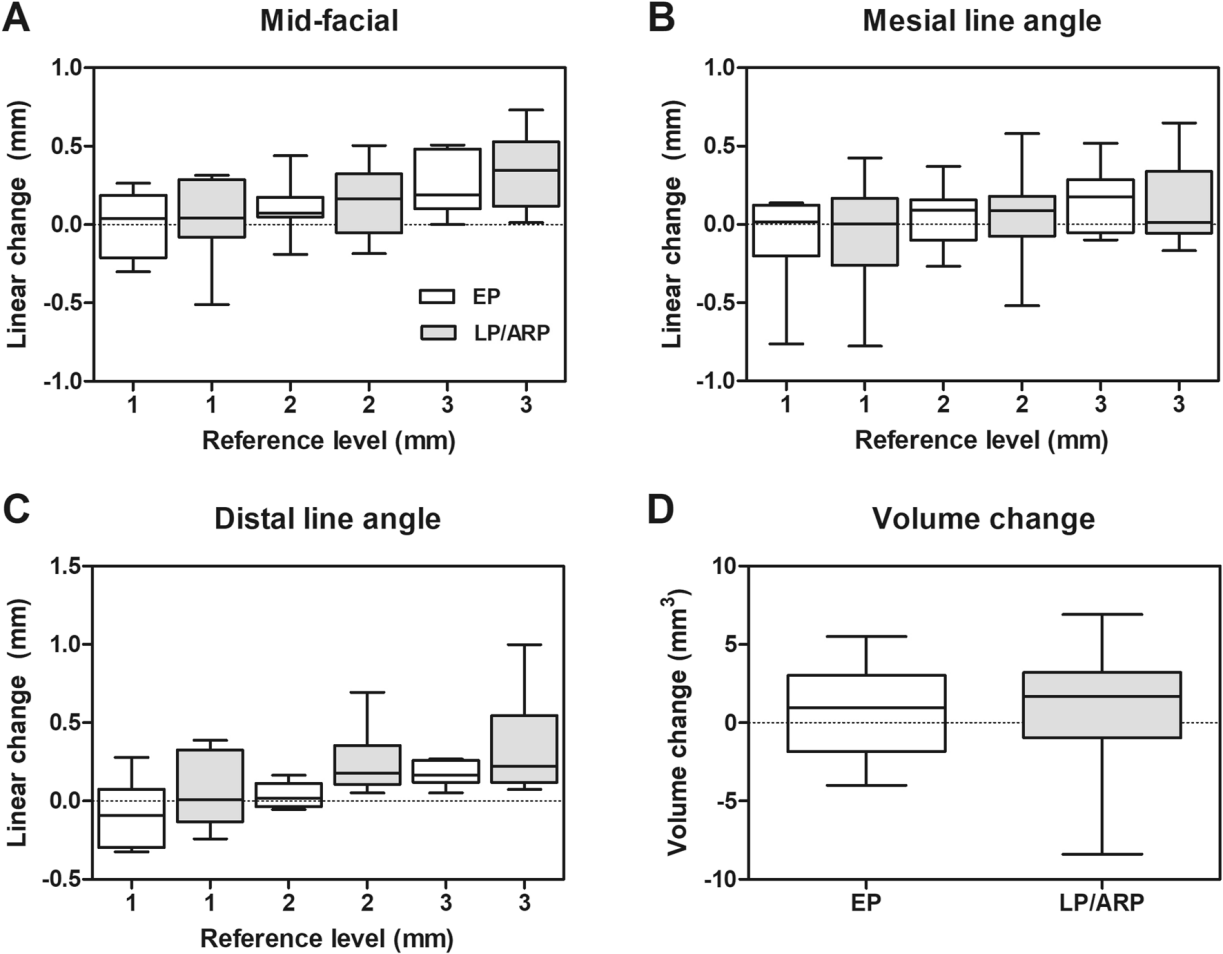
Graphs for linear tissue change and volume change in the area of interest from immediately after the final prosthesis insertion and after 12 months. (**a**) Linear difference at the mid-facial line. (**b**) Linear difference at the mesial line angle. (**c**) Linear difference at the distal line angle. (**d**) Volume difference. Positive and negative numbers indicate gain and loss, respectively. The whiskers cover the whole range of the data.

At all levels of the mid-facial line in both groups, the median values of the changes demonstrated an increase of the tissue dimension (ranging between 0.04 mm [− 0.05, 0.26] and 0.35 mm [0.12, 0.52 mm] in group LP/ARP and between 0.04 mm [− 0.18, 0.15] and 0.19 mm [0.11, 0.36] in group EP). These changes were more significant in the apical direction. In all comparisons between the two groups, no statistically significant differences were observed (*p* = 0.408, *p* = 0.758 and *p* = 0.408 at 1 mm, 2 mm and 3 mm level, respectively) (Table 2; Fig. 3a).

The data at the mesial and distal line angles are presented in the supplement 2 and Fig. 3b, c.

### Volumetric changes over time

The median AOI volume increased from baseline (final prosthesis insertion) to 3 months, to 6 months, and to 12 months (1.70 mm^3^ [0.36, 2.0]) in group LP/ARP. In group EP, there was a decrease in the AOI volume from baseline to 3 and 6 months. However, at 12 months, the volume was higher compared to the baseline (0.96 mm^3^ [− 0.55, 2.64]). There were no statistically significant differences between the two groups at all time points (*p* = 0.681, *p* = 0.606 and *p* = 1.0 for T0–T3, T0–T6 and T0–T12, respectively) (Table 3; Fig. 3d).

**Table 3:** Volume changes on a soft tissue level.

Time-point	T3–T0		T6–T0		T12–T0	
Group	LP/ARP	EP	LP/ARP	EP	LP/ARP	EP
Volume change	1.36 ± 3.19	1.02 ± 3.94	0.84 ± 3.52	0.05 ± 4.37	0.88 ± 4.31	0.95 ± 3.14
	3.00 (− 0.88, 3.37)	− 0.81 (− 1.4, 2.78)	1.03 (0.06, 1.45)	− 1.19 (− 1.79, 3.15)	1.7 (0.36, 2.0)	0.96 (− 0.55, 2.64)
*p* value	0.681		0.606		1	

Data are presented as a mean ± standard deviation, median (1st quartile, 3rd quartile). Positive and negative numbers indicate gain and loss, respectively. LP/ARP: late implant placement following 4 months of alveolar ridge preservation, EP: early implant placement. There were no statistically significant differences between the two groups.

## Discussion

The present study three-dimensionally compared late implant placement following ARP (group LP/ARP) and early implant placement (group EP) in periodontally compromised extraction sites on the soft tissue level. It was demonstrated that (1) linear tissue contour changes between the final prosthesis insertion and after 12 months were minimal in both groups (< 0.5 mm; 0.04–0.35 mm in group LP/ARP, 0.04–0.19 mm in group EP), (2) there was an increase in volume in both groups (1.7 mm^3^ in group LP/ARP, 0.96 mm^3^ in group EP), and (3) there was no statistically significant difference in the above parameters between the two groups. Therefore, the null hypothesis of the present study was not rejected, which indicated that there was no significant difference between groups LP/ARP and EP in terms of the profilometric (linear and volumetric) changes on the soft tissue level for periodontally compromised non-molar extraction sites.

Extensive research exists on tissue stability following various implant treatment modalities. However, two-dimensional measurements were mostly used to obtain tissue changes. For example, the changes of mucosal margin and papilla were assessed using periodontal probe or caliper on study casts, clinical photographs, or directly on the patients^10–12^. Furthermore, three-dimensional information is insufficient. Most of the data have been derived from specific treatment modalities (mainly regarding immediate implant placement)^13,14^ and limited time points (for instance in ARP, between the time of ARP and implant placement)^15^. To the best knowledge, the present study is the first to report the profilometric changes measured three-dimensionally at sites treated with either LP following ARP or with EP.

Based on the data of the present study, there is some gain or loss of the tissue over time in both groups. However, the tissue contour appeared to be stable in the mid-facial area. The linear changes at 3, 6, and 12 months were less than 0.5 mm with respect to the baseline (immediately after final prosthesis insertion) in all levels of the mid-facial line along with the implant prosthesis. The AOI volume measurement also revealed some changes over time, but the net change in tissue thickness increased at T12 in both groups. Those results supported our previous study reporting vertical changes in the marginal tissues^8^. In that study, the median changes of the mid-facial mucosal margin were 0.03 mm in group LP/ARP and − 0.19 mm in group EP between T0 and T12. Taken together, in periodontally compromised extraction sites, LP/ARP and EP protocols yielded stability on the soft tissue level after final prosthesis insertion up to 12 months.

In few recent clinical studies^16,17^, three-dimensionally assessed tissue changes on the soft tissue level around dental implants were investigated. In one study, late implant placement on a single tooth gap in the anterior or premolar region was performed with/without GBR^16^. Tissue changes on the soft tissue level were collected immediately before implant placement and after 3 years. When looking at the data in GBR-treated sites after final prosthesis insertion, there was a slight loss of linear tissue thickness. These changes were between − 0.1 mm (median value) and − 0.2 mm at 6 months and between − 0.3 mm and − 0.5 mm at 30 months after final prosthesis insertion. In another study, two different types of the implant system were compared between the baseline and 5 years^17^. In that study, implant timing was not specified, and bone grafting was performed if necessary. The changes of the tissue thickness were between − 0.15 mm (median value) and − 0.32 mm at 1 year and between − 0.45 mm and − 0.66 mm at 5 years. Volumetric changes ranged between − 0.07 mm and − 0.16 mm at 1 year and between − 0.39 mm and − 0.4 mm at 5 years.

Compared to the above changes, groups EP and LP/ARP in the present study lost more or less a similar tissue thickness at 6 months. At 12 months, two groups demonstrated a gain of tissue volume (between 0.04 mm and 0.16 mm linearly at the 1-mm/2-mm levels, between 0.96 and 1.70 mm^3^ volumetrically), which had a relatively different tendency. Despite some differences among the studies, such measured values seem to be clinically negligible, indicating tissue stability after final prosthesis insertion. Nonetheless, it should be borne in mind that the different amount of tissue change may be derived from different implant treatment modalities with varying implant systems and the resultant tissue remodeling.

In the present study, the tissue changes between extraction and final prosthesis insertion were not recorded. The relevant information may be partially suspected in one preclinical study^18^ and the above clinical study^16^. In the preclinical study testing early implant placement at ARP-treated sites, ARP sites lost 24.8%–32.6% width on the soft tissue level between ARP and 12 weeks following implant placement. In the clinical study, GBR (0.8–0.95 mm) and abutment connection (0.6–0.85 mm) mostly contributed to an increase in tissue volume over 3 years of follow-up. However, it should be noted that the results of the above studies were derived from intact extraction sites^18^ and healed sites^16^. Between the periodontally compromised extraction site and other sites, tissue dynamics manifested on the soft tissue level might differ concerning implant placement, uncovering surgery, and soft tissue molding with a provisional restoration. Currently, no comparative data is available, though.

Peri-implant tissue undergoes constant remodeling process. Thus, profilometric soft tissue change may be an important parameter to evaluate the success of the dental implant^17^. For example, based on the serial studies regarding immediate implant placement, progressive deterioration of the soft tissue level was observed over time^19,20^. In the present study, 1-year outcomes have been presented. The tissue remodeling of the study groups should be constantly monitored with clinical photographs and intra-oral scanning.

From a methodologic point of view, one can argue the influence of alginate impression material even though the analysis itself can be considered to be highly precise. Alginate impression is sensitive to storage conditions and storage time before pouring, which may hamper dimensional stability. Thus, the manufacturer’s guideline of alginate material was strictly followed in the present study to minimize the error. Still, there is a necessity for taking potential error into account, owing to the small linear and volumetric change presented in this study. According to literature, the expected errors derived from alginate impression were as follows: between − 0.1 and − 0.48% for cross-arch measurement, between − 0.92 and 0.48% for anterior–posterior measurement (from the labial surface of the central incisor to the distal surface of the first molar)^21^, 0.3 mm ± 0.15 mm for inter-abutment distance (a tooth shape)^22^, between 0.06 and 0.16% for a single abutment (a cylindrical shape with a diameter of 6.35–8.89 mm), and between 0.02 and 0.6% for inter-abutment distance^23^. Considering that the measurement in the present study was performed for single tooth gap, it could be suspected the potential error from alginate impression may be within 0.2%^23^. In addition to this, the model scanner may produce an error up to 0.01 mm^24^. Taken together, it can be assumed that potential error in the present methodology might be approximately within 0.03 mm (considering the average value of the crown of non-molar teeth^25^). Such amount reasonably leaves a room for discussion regarding over-accuracy from the hundredths place value. Furthermore, this indicated that complete digital flow is required in further studies even though the present methodology was used in several studies with a similar topic to the present study^17,26–28^.

Other limitations are as follows. First, it was difficult to quantify the actual effect of ARP on tissue stability due to additional bone augmentation at the time of implant placement. However, further bone augmentation should be performed for ethical reasons, compensating the limitation of ARP. Second, the sample size was small. However, the present results could serve as reference data for further clinical trials involving a larger sample size.

## Conclusion

The present case series study investigated the profilometric changes on the soft tissue level for periodontally compromised non-molar extraction sites treated with late implant placement following alveolar ridge preservation (group LP/ARP) and early implant placement (group EP). The results suggested that two treatment modalities could provide similar tissue stability on the soft tissue level over 1 year of prosthetic loading. The present study also indicated the tissue change was minimal to a clinically negligible extent. Based on the present study results, clinical trials on this matter can be further implemented for a higher level of scientific evidence. Moreover, the data of other implant treatment modalities should be collected.

## Supplementary Information

Supplementary Information 1.

Supplementary Information 2.

Supplementary Information 3.

## Data Availability

Raw data are presented in the supplement 3.
