# Supplementary material for: A case series of profilometric changes in two implant placement protocols at periodontally compromised non-molar sites
Source: Sci Rep. 2021 Jan 18;11:1714. doi: 10.1038/s41598-021-81402-5 (PMC7813861; doi:10.1038/s41598-021-81402-5)
Supplement: Supplementary file 1 — Supplementary Information 1. [file 41598_2021_81402_MOESM1_ESM.docx]

**A case series of profilometric changes in two implant placement protocols at periodontally compromised non-molar sites**

Kwantae Noh^1^, Daniel S. Thoma^2^, Jung-Chul Park^3^, Dong-Woon Lee^4^, Seung-Yun Shin^5^, Hyun-Chang Lim^5*^

**Authors’ affiliation:**

^1^Department of Prosthodontics, School of Dentistry, Kyung Hee University, Seoul, Republic of Korea

^2^Clinic of Reconstructive Dentistry, University of Zurich, Zurich, Switzerland

^3^Department of Periodontology, College of Dentistry, Dankook University, Cheonan-si, Republic of Korea

^4^Department of Periodontology, Veterans Health Service Medical Center, Seoul, Republic of Korea

^5^Department of Periodontology, Periodontal-Implant Clinical Research Institute, School of Dentistry, Kyung Hee University, Seoul, Republic of Korea

**Running title:** Profilometric change after implant placement

**Corresponding author:**

*Hyun-Chang Lim

Department of Periodontology, Periodontal-Implant Clinical Research Institute, School of Dentistry, Kyung Hee University, 26 Kyungheedae-ro, Dongdaemun-gu, Seoul 02447, Republic of Korea

Tel: +82-2-958-9382, Fax: +82-2-958-9387, E-mail: *Hyun-Chang.Lim@khu.ac.kr*

**Supplement 1. The information of the previous study providing the data**

**Study title**

Late implant placement following ridge preservation versus early implant placement: A pilot randomized clinical trial for periodontally compromised non-molar extraction sites. *J Clin Periodontol* 47, 247-256

**Null hypothesis**

One of the implant placement modailities (LP/ARP or EP) was inferior to the other one in terms of mid-facial mucosal margin for periodontally-compromised non-molar extraction sites.

**Sample size calculation**

G Power software was used for sample size calculation. The change of the midfacial mucosal level (0.6 mm) following early implant placement was used for the reference. A change of >1.0 mm of the mucosal level was considered a clinically significant change. Standard deviation was set to 0.6 mm. To obtain 80% power at a two-sided alpha level of 0.05%, at least seven patients per group were required (allocation ratio 1:1).

**Registration**

The protocol was registered at the Korean Clinical Research Information Service (CRIS; KCT0004014). The present study was registered in the CRIS (30, May 2019).
