# Supplementary material for: A case series of profilometric changes in two implant placement protocols at periodontally compromised non-molar sites
Source: Sci Rep. 2021 Jan 18;11:1714. doi: 10.1038/s41598-021-81402-5 (PMC7813861; doi:10.1038/s41598-021-81402-5)
Supplement: Supplementary file 2 — Supplementary Information 2. [file 41598_2021_81402_MOESM2_ESM.docx]

**A case series of profilometric changes in two implant placement protocols at periodontally compromised non-molar sites**

Kwantae Noh^1^, Daniel S. Thoma^2^, Jung-Chul Park^3^, Dong-Woon Lee^4^, Seung-Yun Shin^5^, Hyun-Chang Lim^5*^

**Authors’ affiliation:**

^1^Department of Prosthodontics, School of Dentistry, Kyung Hee University, Seoul, Republic of Korea

^2^Clinic of Reconstructive Dentistry, University of Zurich, Zurich, Switzerland

^3^Department of Periodontology, College of Dentistry, Dankook University, Cheonan-si, Republic of Korea

^4^Department of Periodontology, Veterans Health Service Medical Center, Seoul, Republic of Korea

^5^Department of Periodontology, Periodontal-Implant Clinical Research Institute, School of Dentistry, Kyung Hee University, Seoul, Republic of Korea

**Running title:** Profilometric change after implant placement

**Corresponding author:**

*Hyun-Chang Lim

Department of Periodontology, Periodontal-Implant Clinical Research Institute, School of Dentistry, Kyung Hee University, 26 Kyungheedae-ro, Dongdaemun-gu, Seoul 02447, Republic of Korea

Tel: +82-2-958-9382, Fax: +82-2-958-9387, E-mail: *Hyun-Chang.Lim@khu.ac.kr*

**Supplement 2. Linear tissue changes at mesial/distal line angles on soft tissue levels**

|  | Group LP/ARP | | | Group EP | | |
| --- | --- | --- | --- | --- | --- | --- |
| Mesial line angle | | | | | | |
|  | T3-T0 | T6-T0 | T12-T0 | T3-T0 | T6-T0 | T12-T0 |
| 1 mm | -0.05 ± 0.27  -0.01 (-0.14, 0.13) | -0.05 ± 0.31  -0.03 (-0.26, 0.25) | -0.06 ± 0.35  0, (-0.25, 0.15) | -0.09 ± 0.28  -0.07 (-0.27, 0.08) | -0.18± 0.34  -0.09 (-0.26, 0.03) | -0.12 ± 0.32  0.01 (-0.19, 0.09) |
| 2 mm | 0.06 ± 0.22  0.14 (-0.03, 0.17) | 0.06 ± 0.24  0.03 (-0.08, 0.14) | 0.06 ± 0.29  0.09 (-0.02, 0.16) | 0.07 ± 0.26  0.06 (-0.14, 0.25) | - 1. ± 0.26   0.02 (-0.08, 0.14) | 0.05 ± 0.2  0.09 (-0.05, 0.14) |
| 3 mm | 0.14 ± 0.21  0.07 (-0.04, 0.38) | 0.17 ± 0.22  0.12 (0, 0.37) | 0.13 ± 0.26  0.01 (-0.03, 0.26) | 0.16 ± 0.27  0.11 (-0.03, 0.25) | 0.1 ± 0.25  0.11 (-0.03, 0.26) | 0.18 ± 0.21  0.18 (0.05, 0.28) |
| Distal line angle | | | | | | |
|  | T3-T0 | T6-T0 | T12-T0 | T3-T0 | T6-T0 | T12-T0 |
| 1 mm | 0.13 ± 0.2  0.18 (0, 0.31) | 0.07 ± 0.26  -0.06 (-0.15, 0.35) | 0.07 ± 0.23  0.01 (-0.12, 0.31) | -0.04 ± 0.2  -0.15 (-0.19, 0.13) | -0.07 ± 0.32  -0.16 (-0.21, 0.1) | -0.06 ± 0.21  -0.09 (-0.2, 0.05) |
| 2 mm | 0.21 ± 0.16  0.21 (0, 0.31) | 0.21 ± 0.25  0.19 (0, 0.28) | 0.25 ± 0.2  0.18 (0.14, 0.34) | -0.02 ± 0.17  -0.09 (-0.14, 0.08) | -0.01 ± 0.21  0.02 (-0.17, 0.16) | 0.04 ± 0.08  0.02 (-0.01, 0.09) |
| 3 mm | 0.32 ± 0.25  0.31 (0.11, 0.45) | 0.38 ± 0.26  0.28 (0.24, 0.42) | 0.34 ± 0.3  0.22 (0.14, 0.52) | 0.11 ± 0.22  0.01 (-0.02, 0.2) | 0.07 ± 0.2  -0.02 (-0.07, 0.25) | 0.18 ± 0.08  0.16 (0.13, 0.25) |

Data are presented mean ± standard deviation, median (1^st^ quartile, 3^rd^ quartile).

Group LP/ARP: late implant placement following 4 months alveolar ridge preservation, Group EP: early implant placement. There were no statistical significantly difference between two groups at all levels.
